# Antiproliferative and pro-apoptotic effects afforded by novel Src-kinase inhibitors in human neuroblastoma cells

**DOI:** 10.1186/1471-2407-10-602

**Published:** 2010-11-04

**Authors:** Michele Navarra, Marilena Celano, Jessica Maiuolo, Silvia Schenone, Maurizio Botta, Adriano Angelucci, Placido Bramanti, Diego Russo

**Affiliations:** 1 Pharmaco-Biological Department, University of Messina, viale Annunziata, 98100, Messina, Italy; 2 Department of Pharmacobiological Sciences, University "Magna Graecia" of Catanzaro, viale Europa, 88100, Catanzaro, Italy; 3 Department of Pharmaceutical Sciences, University of Genova, viale Benedetto XV, 16132 Genova, Italy; 4 Pharmaco-Chemical-Technological Department, University of Siena, via Aldo Moro 2, 53100, Siena, Italy; 5 Department of Experimental Medicine, University of L'Aquila, via Vetoio, 67100 Coppito, L'Aquila, Italy; 6 IRCCS centro neurolesi "Bonino-Pulejo", via Palermo C/da Casazza, 98124, Messina, Italy

## Abstract

**Background:**

Neuroblastoma (NB) is the second most common solid malignancy of childhood that usually undergoes rapid progression with a poor prognosis upon metastasis. The Src-family tyrosine kinases (SFKs) are a group of proteins involved in cancer development and invasiveness that seem to play an important role in the NB carcinogenesis.

**Methods:**

To determine cell proliferation, the growth rate was evaluated by both MTT test and cells counted. Analysis of DNA content was performed for the evaluation of the cell cycle and apoptosis. To characterize the mechanisms underlying the antiproliferative effects induced by SI 34, a novel pyrazolo-pyrimidine derivative provided with Src inhibitory activity, the involvement of some cellular pathways that are important for cell proliferation and survival was investigated by western blot assays. In particular, the contribution of cyclins, Src and ERK were examined. Finally, experiments of cell adhesion and invasiveness were performed.

**Results:**

Treatment of SH-SY5Y human NB cells and CHP100 human neuroepithelioma (NE) cultures with three novel pyrazolo[3,4-*d*]pyrimidine derivatives, namely SI 34, SI 35 and SI 83, inhibits the cell proliferation in a time and concentration-dependent manner. The maximal effect was obtained after 72 hours incubation with SI 34 10 μM. Fluorescence microscopy experiments, flow cytometry analysis and determination of caspase-3 activity by fluorimetric assays showed that SI 34 induced SH-SY5Y apoptosis. Moreover, SI 34 determined cell cycle arrest at the G0/G1 phase, paralleled by a decreased expression of cyclin D1. Furthermore, our data indicate that SI 34 reduces the SH-SY5Y cells adhesion and invasiveness. Evidence that SI 34 inhibits the Src and the ERK-phosphorylation, suggests the mechanism through which it exerts its effects in SH-SY5Y cells.

**Conclusions:**

Our study shows the ability of this pyrazolo-pyrimidine Src inhibitor in reducing the growth and the invasiveness of human NB cells, suggesting a promising role as novel drug in the treatment of neuroblastoma.

## Background

Neuroblastoma (NB) is the most common extracranial pediatric solid tumour. It accounts for more than 7% of malignancies in patients younger than 15 years and around 15% of all paediatric oncologic deaths. NB originates from neural crest precursor cells as the results of genetic alterations occurring in neural crest cells that affect the normal developmental program [1,2]. NB may present with a broad spectrum of clinical behaviour and may have various prognosis depending on the assignment to a risk group. However, about half of patients present with evidence of metastasis and the majority of tumors usually undergo rapid progression with a fatal outcome. Although an aggressive and intensive multimodality approach (surgery, cytotoxic chemotherapy, radio-metabolic treatment) has produced some improvements in the overall cure rate of these patients, the treatment strategies are still far from satisfaction [1,2]. Thus, innovative drugs are needed to develop novel therapeutic strategies acting to ameliorate the prognosis of NB patients.

Several studies have identified the protein tyrosine kinases (TKs) as targets for cancer therapy, since enhancement of TK activity has been correlated with cancer and other proliferative diseases [3]. For this reason, many TK inhibitors (TKIs) have been tested for their *in vitro *and *in vivo *anticancer activity [4], and some of them have been approved in clinical trials or are in clinical use [5,6]. A subclass of TKIs with strong antiproliferative activity is represented by the inhibitors of Src-family tyrosine kinases (SFK), a group of non-receptor TKs involved in cancer development and invasivity [7,8]. Src can stimulate cell proliferation, migration and invasion as well as angiogenesis [9]. Moreover, recent studies have suggested that Src may be implicated in the development of drug resistance [10]. Over-expression or aberrant activation of Src has been detected in a variety of human cancers [11], including NB [12,13], thus representing an attractive target for therapeutic strategies against this tumour.

In the last years a series of novel pyrazolopyrimidine derivatives synthesized in our laboratory have been found to be able to inhibit Src phosphorylation and to exert a potent antiproliferative action on different human carcinoma cells, including A431 (epidermoid) and 8701-BC (breast cancer) cell lines overexpressing Src. Moreover the compounds reduce proliferation, migratory ability and adhesive capacity of the invasive prostate carcinoma cell line PC3 and inhibit the growth of various human thyroid cancer cell lines. Some terms of the pyrazolo-pyrimidine series showed antiproliferative activity on human osteogenic sarcoma (SaOS-2) cells, reducing bone resorption when used to treat mouse osteoclast and importantly decreased the volume of human SaOS-2 xenograft tumour model in nude mice [14-19]. Very recently we also showed that the compounds are able to greatly reduce the growth rate of medulloblastoma cells by decreasing Src phosphorylation and to inhibit tumour growth *in vivo *in a medulloblastoma mouse model [20].

In this work, we describe for the first time that micromolar concentration of pyrazolopyrimidine derivatives reduce SH-SY5Y human neuroblastoma cells survival and invasion, suggesting a potential role as novel drugs in neuro-oncology.

## Methods

### Drugs

SI 34 and SI 35 were synthesized as previously described [14,15]. SI 83 was synthesized in a similar way, but performing the last step with meta-chloro-aniline in ethanol at reflux [16]. Stock solutions of each compound were prepared in dimethylsulfoxide (DMSO) at 50 mM, stored in aliquots at -20°C and diluted in culture media to the desired concentration just prior to use. The maximal concentration of DMSO utilized in this study (0.02% in culture media, corresponding to the concentrations in 10 μM dilution of SI compounds) served as vehicle controls. In comparison with untreated cultures, DMSO 0.02% (1:5000 dilution) did not exert any significant influence on any parameters analyzed in this study (data not shown).

### Cell culture, proliferation assays and cytotoxicity study

Experiments were carried out using two different human peripheral nervous system (PNS) tumour cell lines, the CHP100 human neuroepithelioma (NE) and the SH-SY5Y human neuroblastoma (NB) culture [21,13] that were grown as described [22,23]. To determine cell proliferation, the cultures were seeded onto 6-well plates (100×10^3 ^cells/well) for cell count or 96-well plates (5×10^3 ^cells/well) for MTT assay. On the next day, the growth medium was replaced with fresh medium (untreated cultures) or with medium containing the pyrazolopyrimidine derivatives ranging from 1 to 10 μM. Then, the cell growth was evaluated spectrophotometrically (MTT test) or by cells counted after 24, 48 and 72 hour incubation [19].

Cytotoxicity was assessed by the trypan blue dye exclusion test [22]. All reagents were from Sigma-Aldrich (Milan, Italy).

### Cytofluorimetric analysis

Analysis of DNA content was performed for the evaluation of the cell cycle. 150×10^3 ^SH-SY5Y cells were plated in 35 mm dishes and treated the next day with SI 34 (1 and 10 μM) for 24-72 h. After stimulation, SH-SY5Y cells were collected by trypsinization and centrifuged for 5 min at 200 g. Then, the cells were fixed in cold 70% ethanol at 4°C for 2 hours, resuspended in 500 μl of staining solution (40 μg/ml propidium iodide and 500 μg/ml RNase A in PBS; all from Sigma) for 30 min at 37°C and analyzed by flow cytometry.

Annexin V staining was performed according to the kit manufacturer's instructions (BD Biosciences, Milan, Italy) to detect the apoptosis. Briefly, the cells were detached by trypsin, washed with cold PBS, and suspended in 1× binding buffer at a concentration of 1×10^6 ^cells/ml. Hundred microliters of the suspension were transferred to a 5 ml culture tube and 5 μl FITC Annexin V were added. The samples were gently vortexed and incubated for 15 min at 25°C in the darkness. Finally, 400 μl of 1× binding buffer were added to each tube and the samples were analyzed by flow cytometry within 1 hour. A FACSCalibur (BD Biosciences) flow cytometer was used and the analysis was performed with FlowJo software (BD Biosciences). Cultures treated with etoposide (50 μM, Sigma-Aldrich) were used as positive control, both in cell cycle analysis and apoptosis detection. Three sets of 10000 events were collected for each condition.

### Analysis of nuclear morphology by fluorescence microscopy

SH-SY5Y cells were plated on glass coverslips and treated with 1-10 μM SI 34 for 24-72 hours. Then, the cultures were fixed with 2% paraformaldehyde for 20 min at 37°C and stained with 1 μg/ml of the DNA-binding fluorochrome Hoechst 33258 (Bio-rad Laboratories, Milan, Italy). Finally, the cells were observed with a Nikon Diaphot fluorescence microscopy [24].

### Caspase-3 activity assay

At the end of the SI 34 incubation (1 and 10 μM; 24-72 hours), SH-SY5Y cells were harvested and lysed in ice-cold lysis buffer (50 mM HEPES, pH 7.4, 150 mM NaCl, 5 mM MgCl_2_, 5 mM EDTA, 0.1% 3[(3-cholamidopropyl) dimethylammonium]-1-propanesulfonate (CHAPS), 5 mM dithiothreitol, 10 μg ml^-1 ^pepstatin A, 10 μg ml^-1 ^leupeptin and 10 μg ml^-1 ^aprotinin). The fluorimetric assay for caspase-3 activity was performed as follows [23]. Cell suspensions were sonicated, centrifuged at 12000 g for 10 min at 4°C and protein concentration in supernatants was determined by the DC protein assay (Bio-Rad Laboratories). Cell supernatants were diluted in assay buffer (100 mM HEPES, pH 7.4, 5 mM EDTA, 0.1% CHAPS, 5 mM dithiothreitol and 10% glycerol) to a final concentration of 0.6 μg of protein per μl and incubated in triplicate in a 96-well clear-bottom plate with the fluorogenic substrate acetyl-Asp-Glu-Val-Asp-7-amino-4-methylcoumarin (Ac-DEVD-AMC; 50 μM; Bachem, Bubendorf, Switzerland). Production of fluorescent-free AMC, released by caspase-3 activity, was monitored over 60 min at 37°C using a microplate fluorometer (Victor^2 ^multilabel counter, Perkin-Elmer Life Sciences; excitation, 355 nm; emission, 460 nm). The specific contribution of caspase-3 activity was determined by preincubating parallel sample aliquots with the caspase-3 preferring inhibitor acetyl-Asp-Glu-Val- Asp-aldehyde (Ac-DEVD-CHO; 50 μM; Bachem) for 10 min at 37°C before the addition of the caspase substrate; the difference between the substrate cleavage activity in the absence and presence of Ac-DEVD-CHO was regarded as specific caspase-3 activity.

### Western blot analysis

Total proteins were extracted from SH-SY5Y cells as previously described [19]. Confluent cell cultures from three 100 mm Petri dishes were collected and homogenized in 1 ml of buffer containing 50 mM Tris-HCl, 150 mM NaCl, 1%Triton, 0.25% sodium deoxycholate, 10 mM sodium pyrophosphate, 1 mM NaF, 1 mM sodium orthovanadate, 2 mM PMSF, 10 μg/ml leupeptin, and 10 μg/ml aprotinin (all from Sigma-Aldrich). The homogenate was centrifuged at 10000 g (4°C for 10 min), and the supernatant containing the whole-cell lysate was quantified spectrophotometrically using the Bradford method. Twenty micrograms of proteins were loaded onto a 7.5% SDS-polyacrylamide gel electrophoresis and electrotransferred to a Hybond ECL-PVDF nitrocellulose membrane (GE Healthcare, Milan, Italy). Membranes were blocked with TTBS/milk (TBS, 1% Tween 20, and 5% non-fat dry milk) for 2 hours at room temperature and incubated with the following primary antibodies: a 1:1500 dilution of both an anti-Cyclin D1 and an anti-Cyclin E antibodies (Santa Cruz Biotechnology Inc., Santa Cruz, CA, USA), an anti-Src monoclonal antibody diluted 1/100 or a polyclonal antibody anti-phospho-Src (Cell Signaling Technology, Beverly, MA, USA; cat n. 2102 and 2101, respectively), a 1/2000 dilution of a polyclonal antibody anti-ERK2 or 1/100 of a polyclonal antibody anti-phospho-ERK (Santa Cruz Biotechnology Inc.) and a mouse monoclonal anti-human beta-actin antibody (Sigma-Aldrich) diluted 1:5000 in TTBS/milk. After repeated TTBS washes, the membrane was incubated with horseradish peroxidase-conjugated anti-mouse or anti-rabbit antibody (Transduction Laboratories, Lexington, KY, USA) diluted 1:10000 in TTBS/milk and the protein was visualized with an enhanced chemiluminescence Western blot detection system (ECL plus, GE Healthcare).

### Cell adhesion and invasion assays

Experiments of cell adhesion were conducted in the same condition used in the cell proliferation assay (see above). At the end of the SI 34 incubation times, the morphology of SH-SY5Y cultures was examined by a Nikon Diaphot microscope. Then, the cells were detached by gentle agitation, washed off with culture media, collected and counted in Neubauer hemocytometric chamber in presence of trypan blue dye to distinguish between live and dead cells in suspension. The weakly adherent cells were expressed as percentage of the total cells present in the well, after subtraction of the percentage of dead cells from the full amount of detached cells. The adhesive capacity on specific substrates was assayed by seeding cells at 5×10^5 ^cells/cm^2 ^in a 96-well plate coated with 10 μg/ml of Matrigel or Collagen I (Sigma-Aldrich). Thirty minutes after seeding, adherent cells were fixed with cold methanol for 10 min, washed with phosphate-buffered saline (PBS) and air-dried. Adherent cells were stained with 100 μl of 0.5% crystal violet w/v for 15 min at room temperature. Then cells were rinsed with PBS and lysed with 2% sodium dodecyl sulphate (SDS) w/v, 0.05% sodium azide w/v in water for 1 hour with gentle agitation. Absorbance was measured at 595 nm in a Bio-Rad Multiscan plate reader (Hercules, CA, USA).

Cell invasion experiments were performed with the Matrigel Invasion Chambers constituted by 24 well plates equipped with 8 mm pore size polycarbonate filters overcoated with matrigel (Corning Inc., New York, NY, USA). SH-SY5Y cells (1×10^5^) were seeded in RPMI with 2% FCS in the upper compartment of each chamber. Medium with 10% serum was added to the lower compartment. SI 34 was added to the upper compartment and, after 24, 48 and 72 hours, the cells which had invaded to lower side of the Matrigel coated filter were collected and counted in Neubauer hemocytometric chamber.

### Statistical analysis

Data were expressed as mean ± S.E.M. and statistically evaluated for differences using one-way analysis of variance (ANOVA), followed by Turkey-Kramer multiple comparison test (GrafPAD Software for Science).

## Results

### Structures of pyrazolo[3,4-*d*]pyrimidines

**Figure 1:**
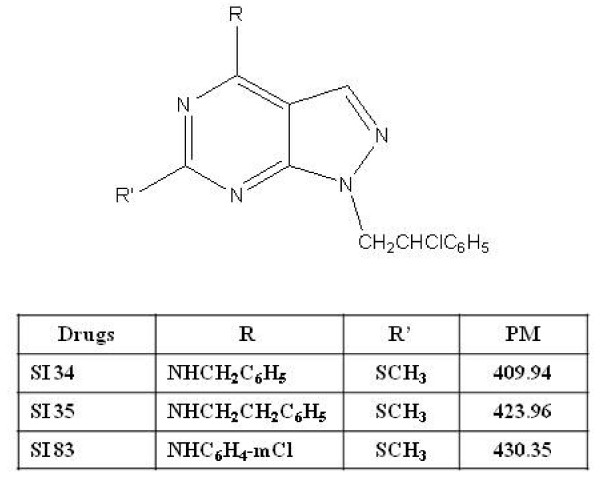
**Chemical structure of the pyrazolopyrimidine derivatives used in this study**.

The 4-amino substituted pyrazolo[3,4-*d*]pyrimidine ring represents a very interesting scaffold for the synthesis of molecules potentially endowed with antitumor activities; this structure is in fact isoster with that of the purine derivative adenine, present in ATP, the natural phosphorylating agent that binds TKs. In our series of derivatives a phenyl ring directly linked with the C4 amino function (anilino derivative SI 83), or spaced by a methylene (benzylamino derivative SI 34) or an ethylene (phenylethylamino derivative SI 35) unit afforded the most active compounds, at least for the biological activities tested until now. Moreover the chlorophenylethyl N1 side chain and the C6 methylthio group are fundamental to maintain both enzymatic and cell activities [14-19]. Structures of SI 34, SI 35 and SI 83 are reported in Figure 1.

### Effects of SI derivates on neuroblastoma and neuroepithelioma cell growth

**Figure 2:**
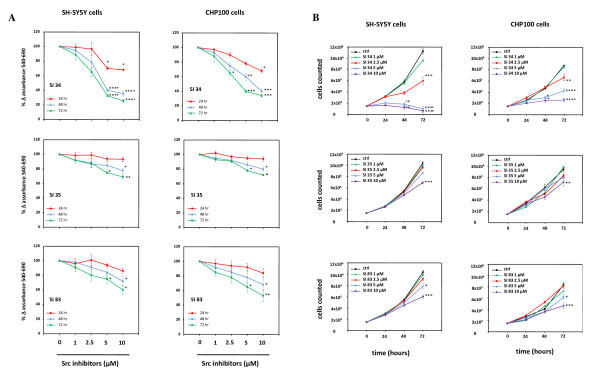
**SI derivatives inhibit SH-SY5Y and CHP100 cell growth**. Both SH-SY5Y and CHP100 cells were exposed to increasing concentration of SI 34 compounds for the indicated periods of time. Proliferation rate was assessed by MTT assay (A) and cell counting (B). Results of MTT test are expressed as percentages of the values detected in untreated cells. Each value is the mean ± S.E.M. of at least three experiments performed in eightplicate (MTT test) or in triplicate (cell counting). *, ** and ***P < 0.05, *P *< 0.01 and *P *< 0.001 *vs *control and SI 34 1 μM; °*P *< 0.05 *vs *SI 34 2.5 μM.

First, we investigated the ability of the three SI derivates tested in this study to inhibit the proliferation of both SH-SY5Y and CHP100 cell lines. As shown in Figure 2, although with different efficacy, all SI molecules reduced cell growth rate in a time and concentration-dependent manner. In particular, the strongest effect on SH-SY5Y was obtained by SI 34 10 μM, reaching its peak of reduction in cell proliferation after 72 hours of treatment (75% growth reduction in the MTT test; *P *< 0.001 *vs *control; Figure 2A). Similar results were observed using CHP100 cells in which 10 μM SI 34 reduced the proliferation by 65% after 72 hours of incubation (*P *< 0.001 *vs *control; Figure 2A). A lower but still significant antiproliferative effect was observed also after treatment of both SH-SY5Y and CHP100 cells with SI 35 and SI 83 (Figure 2A).

The MTT data was confirmed by counting the cells in a Neubauer hemocytometer chamber after treatment with SI molecules. As in MTT experiments, the best inhibitory effect on the proliferation of both SH-SY5Y and CHP100 cell lines was obtained by 10 μM SI 34 (Figure 2B): 72 hours of exposure determined a 94% reduction in cell proliferation of SH-SY5Y (*P <*0.001 *vs *control) and of 71% of CHP100 cells (*P *< 0.001 *vs *control). Again, SI 35 and SI 83 were less effective in reducing NBs cell proliferation (Figure 2B).

Seventy-two hours exposure to 25 μM concentrations, SI 34, SI 35 and SI 83 killed all the cells (data not shown). On the contrary, no significant effect on cell growth was observed with concentrations lower than 1 μM or after shorter incubation times (data not shown).

### Cytotoxic effects induced by SI molecules

**Figure 3:**
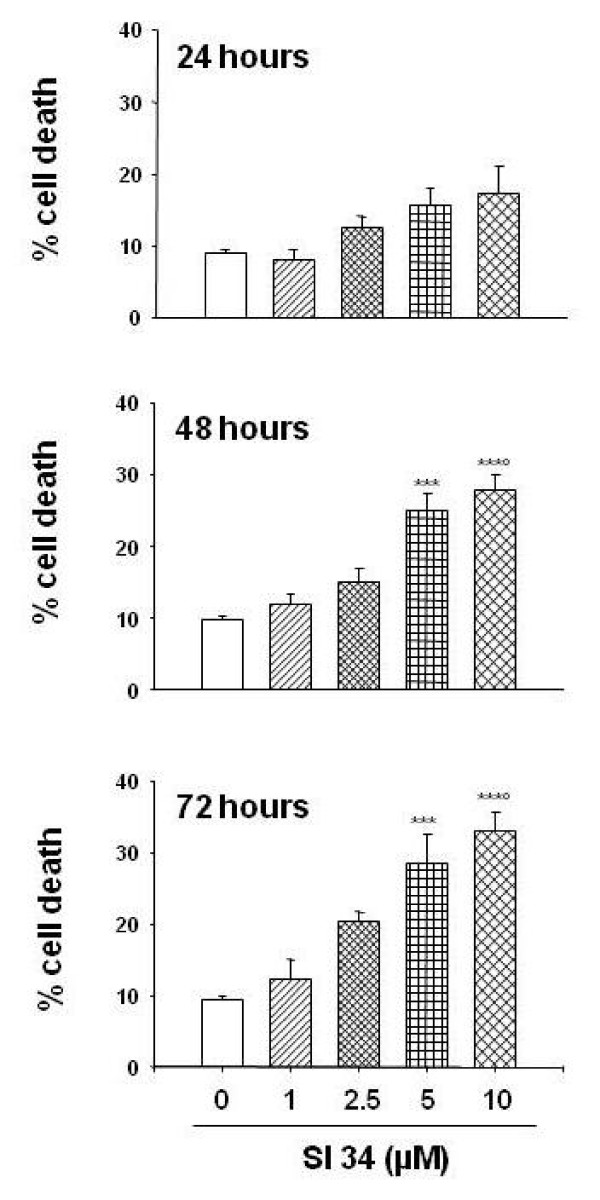
**Cytotoxic effects induced by SI 34 on SH-SY5Y cells**. Cytotoxic effect induced by SI 34 (1-10 μM; 24-72 hours) was evaluated in terms of cell death assessed by trypan blue staining exclusion assay. Data, expressed as mean ± S.E.M., represent the values obtained in three different sets of experiments made in triplicate. ***P < 0.001 *vs *control and SI 34 1 μM; °*P *< 0.01 *vs *SI 34 2.5 μM.

To determine whether SI molecules have cytotoxic effects, both SH-SY5Y and CHP100 cells were exposed to different concentrations of SI 34, SI 35 and SI 83 (1-10 μM) for 24-72 hours, and the cell death was evaluated using the trypan blue dye exclusion assay. As presented in Figure 3, treatment of SH-SY5Y cells with SI 34 resulted in a significant increase in cell death, that rise up to a 33% after 72 hours of incubation (*P *< 0.001 *vs *control). The same trend, but with a low rate of cytotoxicity, was observed treating SH-SY5Y cells with SI 35 and SI 83, and similar results were obtained in CHP100 cells (data not shown). Since the proliferation and cytotoxic analysis revealed that SI 34 was the most active molecule tested in this study, and that the response of CHP100 cells mimicked the effects obtained in SH-SY5Y cells, further studies were carried out testing the action of SI 34 on SH-SY5Y cells only.

### SI 34 induces apoptosis

**Figure 4:**
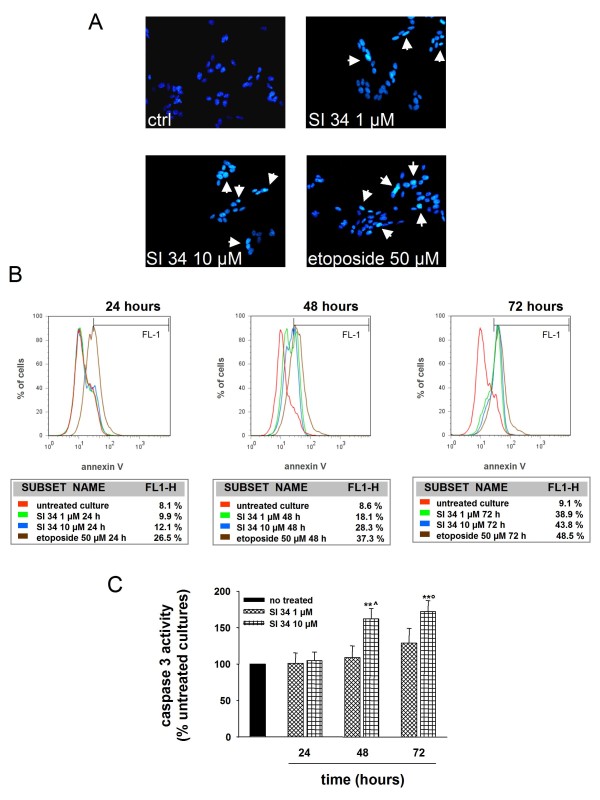
**Evaluation of apoptosis induced by SI 34 on SH-SY5Y cells**. (A) Morphological analysis of nuclear chromatin in SH-SY5Y cells stained with the DNA-binding fluorochrome Hoechst 33258. Cells with morphological changes in the nuclei or condensed chromatin were defined as apoptotic cells. Arrows point the intense nuclear fluorescence present in some cells exposed to both SI 34 (1 and 10 μM) or etoposide (50 μM). Nuclei were visualized by fluorescence microscopy at a magnification of 200×. A representative experiment that was replicated three times with similar results is shown. (B) Annexin V (FITC) staining assay. SH-SY5Y cells undergoing apoptosis were detected by cytofluorimetric analysis as described in materials and methods. Values reported depicted the percentage of events within the gate FL1 used to define AnV+ cells. The red line represents the untreated culture. The green and blue lines symbolize the cells incubated with SI 34 1 and 10 μM, respectively. The brown curve embodies SH-SY5Y cells exposed to etoposide 50 μM as inducer of apoptosis. The FACS analysis shown is representative of three different experiments. (C) Caspase-3 activity assay performed as described in the materials and methods. Results are expressed as percentage of relative fluorescence units (RFU) per min per mg of protein detected in untreated cultures, which are arbitrarily expressed as 100%. Each value represents the mean ± S.E.M of three sets of experiments performed in triplicate. **P < 0.01 *vs *respective control; ^*P *< 0.01 *vs *48 hours SI 34 1 μM; °*P *< 0.05 *vs *72 hours SI 34 1 μM.

To elucidate the type of cell death induced by the SI molecules, several markers of apoptosis were evaluated. We first checked the presence of changes in the morphology of the nuclei by staining the cells with the Hoechst 33258. Apoptotic nuclei were identified by the fragmentation of the nucleus and condensation of nuclear heterochromatin, being highly fluorescent. As illustrated in Figure 4A, after exposure of SH-SY5Y cells to SI 34 (1 and 10 μM) for 72 hours, evidence of apoptotic nuclei was observed.

Apoptosis was also detected by flow cytometric analysis using the Annexin V staining. As shown in Figure 4B, SI 34 10 μM promoted SH-SY5Y cells apoptosis after an exposure of both 48 and 72 hours (28% and 44% of cells, respectively). These results were confirmed by additional experiments aimed to study the cell cycle, in which the fraction of cells in apoptosis was identified as a sub-G0 hypodiploid population (Table 1).

**Table 1:** Cell cycle analysis of SH-SY5Y cells treated with SI 34.

cycle phase	control	SI 34 1 μM	SI 34 10 μM	Etoposide 50 μM
***24 hours treatment***				
**Sub G0**	0	0	0	20 ± 1.2 ***
**G0/G1**	72 ± 1.7	81 ± 2.6	83 ± 1.8 *	69 ± 1.4
**S**	9 ± 0.5	4 ± 0.3 *	3 ± 0.7 *	3 ± 0.2 *
**G2/M**	19 ± 0.7	15 ± 1.0	14 ± 0.9	8 ± 0.8 *
***48 hours treatment***				
**Sub G0**	0	12 ± 1.1 *	24 ± 2.2 ***	44 ± 2.7 ***
**G0/G1**	74 ± 3.1	76 ± 3.5	70 ± 3.6	48 ± 2.6 *
**S**	8 ± 0.8	4 ± 0.5 *	2 ± 0.1 *	1 ± 0.1 **
**G2/M**	18 ± 1.1	8 ± 1.1 *	4 ± 0.1 **	7 ± 0.3 *
***72 hours treatment***				
**Sub G0**	0	42 ± 3.2 ***	45 ± 2.9 ***	80 ± 4.1 ***
**G0/G1**	73 ± 4.7	58 ± 3.2 *	55 ± 2.97 *	20 ± 1.4 ***
**S**	11 ± 1.0	0 ***	0 ***	0 ***
**G2/M**	16 ± 0.8	0 ***	0 ***	0 ***

Progression trough cell cycle was examined by flow cytometry analysis. Data analysis show that SI 34 arrests cell cycle at G0/G1 phase and abolishes the S and G2M phase. Etoposide 50 μM was used as a positive control for cell cycle arrest. Results are represented as percentages of the total cell population and are the mean ± S.E.M. of three independent experiments. *, ** and ***P < 0.05, *P *< 0.01 and *P *< 0.001 *vs *respective control

Additional fluorimetric assays were then performed to verify whether SI 34-mediated apoptosis was associated with caspase-3 activation. A significant increase of caspase-3 activity was observed after 48 and 72 hours of exposure of SH-SY5Y cells to SI 34 10 μM (Figure 4C).

### SI 34 arrests SH-SY5Y cell cycle in G0/G1 phase decreasing the expression of cyclin D1

To better characterize the antiproliferative activity of SI 34, we examined the progression of cells trough the cell cycle by flow cytometry analysis. Table 1 shows that exposure to SI 34 (1-10 μM; from 24 to 72 hours) arrested the SH-SY5Y cell growth in G0/G1 phase in a time and concentration-dependent manner. In particular, the treatment caused a reduction in the G2/M and S phases of the cell cycle that were abolished after 72 hours of incubation, together with a parallel increase in sub G0 phase (Table 1).

**Figure 5:**
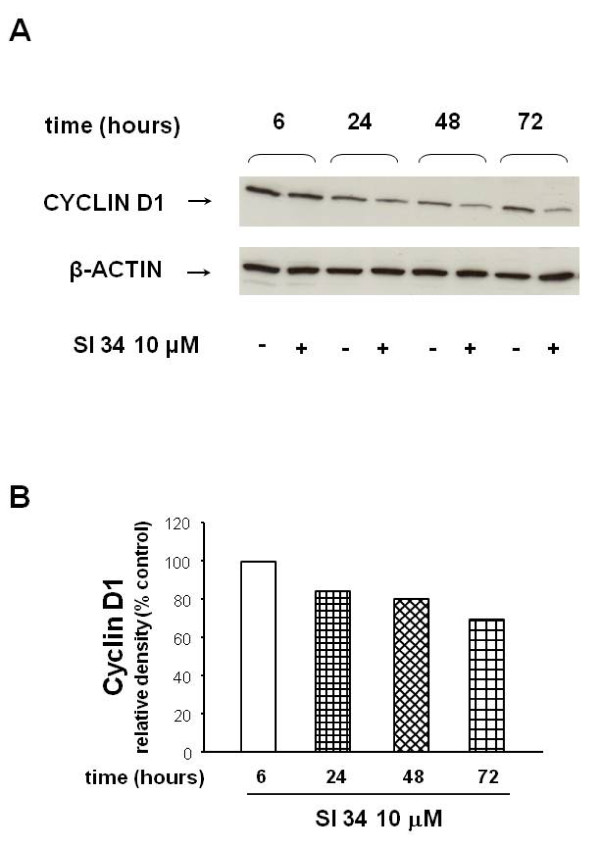
**Analysis of cyclin D1 expression in SH-SY5Y treated with SI 34**. (A) Immunoblot of SH-SY5Y cells exposed to 10 μM SI 34 for 6-72 hours: a representative of three separate experiments is shown. (B) Quantification of cyclin D1 expression from blots in panels A achieved with ImageJ software. Histogram shows the results of the densitometric analysis of autoradiographic bands in which the protein levels were normalized for β-actin (black bars for the untreated cultures and white bars for the cells exposed to SI 34 10 μM for the indicated times). Levels are extrapolated as percentages of the values detected in control cells, which are arbitrarily assigned as 100%.

It is well known the crucial role of the cyclins within cell division cycle and their frequent deregulations in cancer. Cyclin D1 governs the transit through the G1 phase of the cell cycle and is amplified and/or over-expressed in a relevant proportion of human cancers, including NB [25]. In order to estimate the contribution of cyclins in the mechanisms by which SI 34 blocks the SH-SY5Y cell cycle at G0/G1 phase, we examined the expression of cyclin D1 and E in SH-SY5Y cells treated with SI 34 by western blot assays. As shown in Figure 5, when the cultures were exposed to SI 34 10 μM, a time-dependent decrease of the cyclin D1 expression was observed with the maximal reduction detected after 72 h of treatment (*P *< 0.05 *vs *untreated cultures). These data demonstrate that SI 34 is able to reduce the cyclin D1 expression in SH-SY5Y cells, suggesting a correlation between the reduction of this protein level, the cells cycle arrest and the inhibition of cellular proliferation. Cyclin E is another rate limiting regulator in G1 phase of the cell cycle and its appropriate regulation is essential to drive the cells in S phase. Cyclin E appears in late G1 and disappears in early S phase. Interestingly, no modulation of the cyclin E expression by SI 34 was observed (data not shown), strengthening the hypothesis that the block of the cell cycles induced by SI 34 occurs at the early G1 phase.

### SI 34 inhibits Src-TK and ERK-phosphorylation

**Figure 6:**
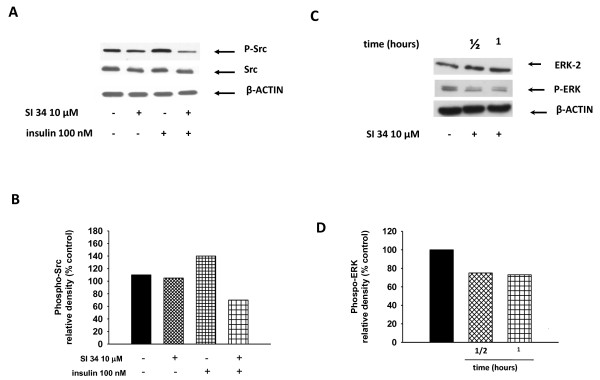
**Src- and ERK-phosphorylation in SH-SY5Y treated with SI 34**. (A) SH-SY5Y cells were stimulated with insulin (100 nM; 30 min) in the presence or absence of SI 34 (10 μM; 30 min), and then western blotting analysis of Src and phospho-Src was performed. A Western blot, representative of three independent experiments showing similar results, is presented. (C) A representative gel (out of three) showed the ERK and phospho-ERK expression in presence or not of SI 34 is illustrated. (B and D) Densitometric analysis of immunoreactive bands corresponding to the Src-phosphorylated and ERK phosphorylated forms from blots A and C are reported. Autoradiographic bands were quantified by ImageJ software and normalized for β-actin levels. Data are reported as percentages of the values detected in untreated cultures.

To better understand the mechanisms underlying the antiproliferative effects induced by SI 34, the involvement of some cellular pathways that are important for cell proliferation and survival like Src and extracellular receptor kinases (ERK) phosphorylation was investigated by western blot assays. In normal conditions, the level of Src-phosphorylation in SH-SY5Y cells is low. Thus, to enhance Src phosphorylation the cells were stimulated with 100 nM insulin for 30 minutes, obtaining an increase of phosphorylated Src level compared to the untreated cultures (Figure 6A and 6B). SH-SY5Y cells stimulated with insulin were treated with SI 34 (10 μM; 30 min) and the levels of phosphorylated and non-phosphorylated Src were examined. As a result, Src-phosphorylation promoted in SH-SY5Y cells by insulin was inhibited using 10 μM SI 34 (*P *< 0.05) while the basal levels of Src were not affected (Figure 6A and 6B).

We next investigated the effects of SI 34 on the phosphorylation of ERK. The results reported in Figure 6C and 6D revealed an early inhibition of ERK-phosphorylation in the SH-SY5Y cells incubated with the test compound (*P *< 0.05 *vs *no treated cells), without any effect on the content of non-phosphorylated protein.

### SI 34 reduces SH-SY5Y adhesion and invasion

**Figure 7:**
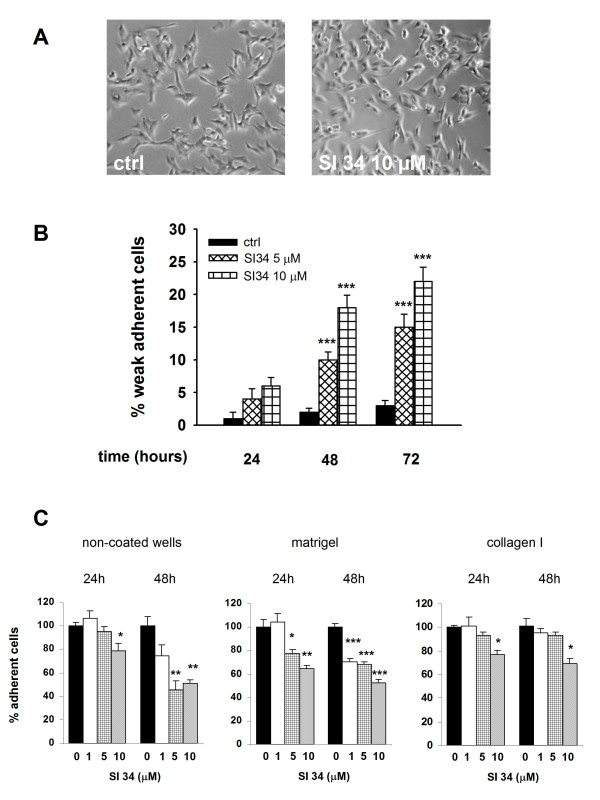
**SI 34 decreases SH-SY5Y cell adhesion**. (A) Changes of cellular morphology in SH-SY5Y cultures exposed to 10 μM SI 34 for 72 hours. (B) Detached cells from cultures exposed (white bars) or not (black bars) to SI 34 were collected and counted as described in materials and methods. The results are expressed as percentage of detached cells (subtracted the percentage of dead cells from the full amount of detached cells) with respect to the total number of cells present in the well. Each value is the mean ± S.E.M. of 6 different sets of experiments made in triplicate. ***P < 0.001 *vs *respective controls. (C) Adhesion assay performed by plating SH-SY5Y cells on two different physiological substrates (Matrigel and collagen I) and on non-coated plastic surface for 30 min. Cells were treated with increasing concentrations of SI 34 (0, 1, 5, 10 μM) for 24 or 48 hours prior the adhesion assay. The values are expressed as mean percentage with respect to control (black bar) of at least three different measurements (± S.E.M). *, ** and ***P < 0.05, *P *< 0.01 and *P *< 0.001 *vs *respective control.

In parallel with the decrease in cell proliferation, we observed that the presence of SI 34 determined a modification in cellular morphology. The cells acquired a round shape morphology, associated with a marked increase of susceptibility in their detachment (Figure 7A). After treatment or not with SI 34, the cells were detached by gentle agitation and counted. The weakly adherent cells reached up to over 20% when the SH-SY5Y cultures were treated for 72 h with 10 μM of the test compound (Figure 7B). Moreover SH-SY5Y cells were treated for 24 and 48 h with increasing concentrations of SI 34 (1, 5 and 10 μM) and then we evaluated their adhesive capacity on two different physiologic substrates, matrigel and collagen I (Figure 7C). The same experimental protocol was executed with non-coated surface. Results demonstrated an evident trend towards a decrease in adhesive capacity of treated cells in presence of all substrates and at higher concentrations of SI 34. In particular, after 48 h, the percentages of adherent cells on matrigel were significantly lower in treated cells than in non-treated cells for all concentrations of SI 34 (-30%, -32%, -47% respectively; *P *< 0.001).

**Figure 8:**
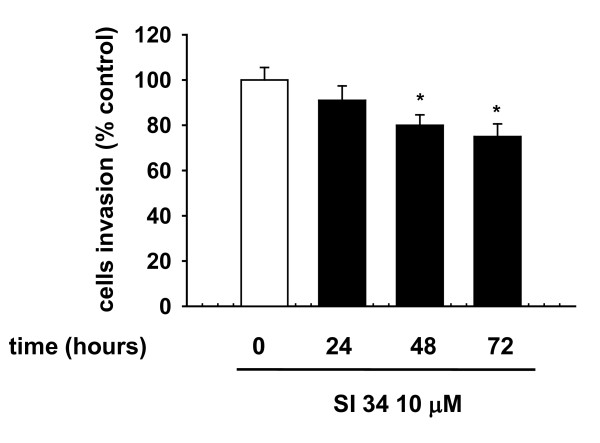
**Reduction of SH-SY5Y invasiveness by SI 34**. Cells treated (filled bar) or not (empty bar) with SI 34 10 μM migrating through the filter of a matrigel Invasion chambers. Data are reported as percentages of cells counted in untreated cultures. Each value represents the mean ± S.E.M. of 3 sets of experiments performed in triplicate. *P < 0.05 *vs *respective controls.

Further studies were focused on the effect of SI 34 on the SH-SY5Y invasion capability. As shown in Figure 8, treatment with 10 μM SI 34 for 24-72 hours reduced the cell invasiveness in a time-dependent manner. The mean number of migrated cells reached statistical significance after 48 and 72 hours incubation with 10 μM of SI 34 (- 20% and - 25% respectively; *P *< 0.05 *vs *control).

## Discussion

Among the novel approaches currently tested against refractory NB, a promising role is played by small molecules with Src inhibitory activity. Indeed, high levels of Src have been found both in specimens from NB, in which correlate with the neuronal/neuroendocrine differentiation, the clinical stage and prognosis, and in NB cell lines such as the SH-SY5Y cells [12,13]. Consistent with these findings, Imatinib, a Bcr-Abl inhibitor, and Dasatinib, a multi-targeted (including Bcr-Abl and Src) inhibitor, have shown growth inhibitory effects on NBs both in *in vitro *and *in vivo *preclinical models [26-29]. Moreover, a phase II clinical trial of Imatinib in children with neuroblastoma and other solid tumors is ongoing [30]. During the past decade, many pyrazolo[3,4-*d*]pyrimidine compounds have been described and reviewed in the literature as selective inhibitors of Src family and as anti-proliferative agents [31]. However, they have never been tested so far against paediatric solid tumours. For the first time, we describe the effects of pyrazolopyrimidine derivatives on human NB cells. In this study we tested three pyrazolo[3,4-*d*]pyrimidine derivatives that have already demonstrated potent inhibition of Src activation and cell proliferation: SI 35 revealed anti-proliferative activity toward human epidermoid carcinoma A431 cells [15] and human prostatic cell line PC3 [17]; SI 34 and SI 35 inhibited the proliferation of human medullary and follicular thyroid cancer cells [18,19]; SI 83 reduced the growth rate in human osteosarcoma cells [16].

Our present results show that SI molecules, although with different extents, inhibit the growth of both SH-SY5Y human NB and CHP100 human NE cell lines in a time and concentration-dependent manner. NB and NE are two closely related tumors of PNS, ontogenetically related but different in some biochemical markers and clinical features [21]. The striking difference in sensitivity of NB and NE cells to SI molecules may reflect the heterogeneity of the human PNS cell lines [21]. Furthermore, the small variations in the chemical structure of the tested compounds might provoke the difference in their intrinsic activity on the two cell lines. Furthermore, SI 34, the compound with the strongest activity, induces caspase-3 activation that in turn causes SH-SY5Y apoptotic cell death. Apoptosis is a physiological process regulating tissue homeostasis. During the normal foetal and postnatal development of the nervous system, apoptosis may occur spontaneously, because of the physiological neuronal differentiation and activity of the cells. Moreover, there is evidence that increased expression of caspases in NBs is associated with favourable biological features and improved disease outcome [32]. For this reason, the NB presenting in the first age may regress spontaneously, determining a good prognosis [1,33]. On the contrary, delayed activation of normal apoptotic pathways might be an important mechanism promoting tumor survival and growth. For this reason, apoptosis of NB cells has become the goal of many studies using novel therapeutics in preclinical models. Our data also demonstrate that SI 34 determined SH-SY5Y cell cycle arrest at the G0/G1 phase, paralleled by decreased levels of cyclin D1, whose over-expression has been described in many human malignancies with poor prognosis [25]. These results are in line with literature data that described a role of Src kinases in the cell cycle regulation, in particular for the progression through G2/M phase [34]. It is known that also ERK activation plays a role in maintaining the growth and the malignant phenotype of cancer cells. Of interest, there is accumulating evidence that Src may be responsible for the ERK activation in several experimental models [35,36], as well as it has been reported that attenuation of ERK pathway may correlate with a decreased cyclin D1 activity in NB cells [37]. In light of the evidence, our results indicate that the reduced Src phosphorylation induced by SI 34 is associated with a decrease of ERK phosphorylation that in turn may reduce the amount of cyclin D1, arresting the SH-SY5Y cell cycle in G0/G1 phase. A relationship between ERK phosphorylation and apoptosis has been recently reported in SH-SY5Y cells [38]. Increased expression and/or activation of both Src and ERK signalling are also critical for tumour cell adhesion and migration [9,39]. Accordingly, inhibition of these kinases by SI 34 reduced the SH-SY5Y cell adhesion on various substrates and invasiveness. In particular, as demonstrated in a different cell model, SI compounds may impair cell adhesiveness and migration through the inhibition of kinases in focal adhesion complex [17]. Taken together, these data suggest that SI 34 may be effective even in the prevention of NB cancer cells dissemination and metastases development. Indeed, treatment with Src inhibitors currently in clinical trials for a wide range of solid tumours could reduce the incidence of metastases [9].

## Conclusions

Our data demonstrate the ability of the novel pyrazolo[3,4-*d*]pyrimidine compounds in reducing the growth, adhesion and invasiveness of human neuroblastoma cells *in vitro*, suggesting a promising role as novel drugs in the treatment of neuroblastoma.

## Abbreviations

NB: neuroblastoma; NE: neuroepithelioma; PNS: peripheral nervous system; SFKs: Src-family tyrosine kinases; TK: tyrosine kinase; ERK: extracellular receptor kinase

## Competing interests

The authors declare that they have no competing interests.

## Authors' contributions

MN helped perform all the experiments, participated in the design of the study and drafted the manuscript. MC and JM carried out all experiments. SS and MB synthesized the molecules tested in this study. AA executed the adhesion assays. PB helped conceive the study, assisted in the study design and interpretation of the data. DR participated in the design of the study and drafting of the manuscript. All authors read and approved the manuscript.

## Pre-publication history

The pre-publication history for this paper can be accessed here:

http://www.biomedcentral.com/1471-2407/10/602/prepub
